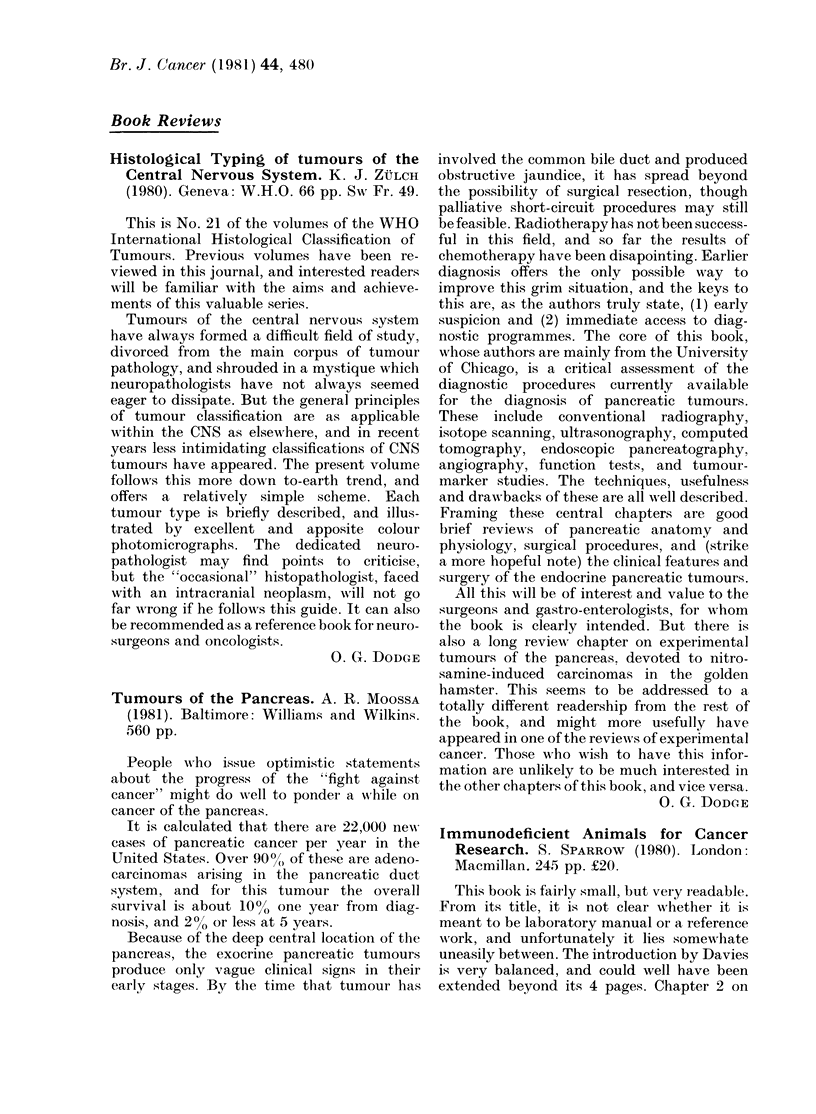# Histological Typing of tumours of the Central Nervous System

**Published:** 1981-09

**Authors:** O. G. Dodge


*Br. J. Cancer* (1981) **44**, 480

### *Book Reviews*

**Histological Typing of tumours of the Central Nervous System.** K. J. Zülch (1980). Geneva: W.H.O. 66 pp. Sw Fr. 49.

This is No. 21 of the volumes of the WHO International Histological Classification of Tumours. Previous volumes have been reviewed in this journal, and interested readers will be familiar with the aims and achievements of this valuable series.

Tumours of the central nervous system have always formed a difficult field of study, divorced from the main corpus of tumour pathology, and shrouded in a mystique which neuropathologists have not always seemed eager to dissipate. But the general principles of tumour classification are as applicable within the CNS as elsewhere, and in recent years less intimidating classifications of CNS tumours have appeared. The present volume follows this more down to-earth trend, and offers a relatively simple scheme. Each tumour type is briefly described, and illustrated by excellent and apposite colour photomicrographs. The dedicated neuropathologist may find points to criticise, but the "occasional" histopathologist, faced with an intracranial neoplasm, will not go far wrong if he follows this guide. It can also be recommended as a reference book for neurosurgeons and oncologists.

O. G. Dodge